# Hyaluronic acid enhances cell migration and invasion via the YAP1/TAZ-RHAMM axis in malignant pleural mesothelioma

**DOI:** 10.18632/oncotarget.20750

**Published:** 2017-09-08

**Authors:** Wataru Shigeeda, Masahiko Shibazaki, Shinji Yasuhira, Tomoyuki Masuda, Tatsuo Tanita, Yuka Kaneko, Tatsuhiro Sato, Yoshitaka Sekido, Chihaya Maesawa

**Affiliations:** ^1^ Department of Tumor Biology, Institute of Biomedical Science, Iwate Medical University, Iwate, Japan; ^2^ Department of Pathology, School of Medicine, Iwate Medical University, Iwate, Japan; ^3^ Department of Thoracic Surgery, School of Medicine, Iwate Medical University, Iwate, Japan; ^4^ Division of Molecular Oncology, Aichi Cancer Center Research Institute, Nagoya, Aichi, Japan

**Keywords:** invasion, migration, mesothelioma, RHAMM, YAP1/TAZ

## Abstract

Most malignant mesotheliomas (MPMs) frequently show activated forms of Yes-associated protein 1 (YAP1) and transcriptional co-activator with PDZ-binding motif (TAZ), which transcriptionally regulates the receptor for hyaluronic acid-mediated motility (RHAMM). As RHAMM is involved in cell migration and invasion in various tumors, we speculated that hyaluronic acid (HA) in pleural fluid might affect the progression of mesothelioma by stimulating cell migration and invasion through RHAMM. The level of RHAMM expression was decreased by YAP1/TAZ knockdown, and conversely increased by forced expression of the active form of YAP1, suggesting that RHAMM was regulated by YAP1/TAZ in MPM cells. Cell migration and invasion were also decreased by YAP1/TAZ or RHAMM knockdown. Notably, HA treatment increased cell motility and invasion, and this was abolished by RHAMM knockdown, suggesting that HA may augment local progression of MPM cells via RHAMM. Furthermore, treatment with fluvastatin, which regulates RHAMM transcription by modulating YAP1/TAZ activity, decreased the motility and invasion of MPM cells. Collectively, these data suggest that HA is an “unfavorable” factor because it promotes malignancy in mesothelioma and that the YAP1/TAZ-RHAMM axis may have potential value as a therapeutic target for inhibition of disease progression in MPM.

## INTRODUCTION

Malignant mesothelioma is an aggressive neoplasm arising from the pleura, pericardium, and peritoneum. It is induced by long-term exposure to asbestos, a naturally occurring silicate mineral, and its incidence and associated mortality rate are increasing in most countries [1, 2]. Although the usage of asbestos is now restricted, it is considered that the incidence of mesothelioma will continue to increase because of the long time taken for mesothelioma to develop following exposure to asbestos, in some cases 15 to 40 years [3, 4]. In many cases, mesothelioma is diagnosed at an advanced stage because of its poor subjective symptoms at the early stage [2]. Among the several types of mesothelioma, malignant pleural mesothelioma (MPM) accounts for approximately 80% of all cases, and poor sensitivity to drug therapy is a frequent clinical problem in affected patients [5]. Despite intensive treatment with surgery, radiation therapy or chemotherapy such as cisplatin and pemetrexed, widely disseminated MPM in the pleural space often spreads to the intra-thoracic lymph nodes, and the clinical outcome is invariably poor [3, 6]. Therefore, a search for molecular targets to inhibit cell migration and invasion would seem justified.

Recent genome-wide studies have revealed that about 50% of MPMs have mutations in Hippo pathway-related genes, especially in *NF2* and *LATS2* [7, 8]. Hippo pathway is known to regulate organ size [9] and tissue homeostasis [10]. Among the several major downstream effectors of Hippo pathway, Yes-associated protein 1 (YAP1) and transcriptional co-activator with PDZ-binding motif (TAZ), a paralog protein of YAP1, have been shown to be attractive candidates for molecular targeted therapy because they regulate many genes involved in cell proliferation, adhesion, and migration [11–14]. Under physiological conditions, stabilization and activation of YAP1/TAZ are tightly regulated by phosphorylation in Hippo pathway [15–17]. Dysregulation of this pathway has been shown to lead to aberrant stabilization and activation of YAP1/TAZ protein, resulting in tumorigenesis, progression, metastasis, and recurrence [18, 19], and further causing drug resistance by acquisition of cancer stem cell-like properties [20–24]. It has been reported that inactivation of LATS2, the major kinase of Hippo pathway, is one of the key mechanisms for aberrant activation of YAP1, and confers a proliferation advantage on MPM cells via transcriptional regulation of cell cycle-related genes such as *CCND1* and *FOXM1* [25].

One of the most notable diagnostic features of MPM is massive pleural effusion containing high levels of hyaluronic acid (HA) [26]. However, the biological relationship between HA and mesothelioma progression still remains to be clarified. The adhesion/homing molecule CD44, which is implicated in cell-cell and cell-matrix adhesion, is the major cell-surface receptor for HA [27]. Recently, it has been reported that YAP1/TAZ and TEAD (TEA domain transcription factor) transcriptional machinery activate CD44 transcription via binding to the CD44 promoter at TEAD-binding sites, thus stimulating the proliferation of MPM cell lines [20]. Beside CD44, receptor for hyaluronic acid-mediated motility (RHAMM, also known as HMMR, IHABP or CD168) functions as a HA receptor [28], and several studies have shown that aberrant expression of RHAMM, which is generally not detected in normal tissues, is involved in cell proliferation, migration, invasion and drug resistance in several tumors including breast [13], lung [29], and liver cancers [30]. Importantly, RHAMM expression is also regulated at the transcriptional level by YAP1/TAZ and TEAD complex via binding at a specific site of the RHAMM promoter, consequently controlling cell migration and invasion in breast cancer cell lines [13]. However, little is known about the contribution of RHAMM to MPM progression. Taking these observations into consideration, we speculated that HA in pleural effusion may promote progression of MPMs by stimulating the YAP1/TAZ-RHAMM axis.

Therefore, we investigated whether HA in pleural effusion could promote cell migration and invasion through the YAP1/TAZ-RHAMM axis in MPMs, and assessed the effects of statin compounds such as fluvastatin, which regulate RHAMM transcription by modulating YAP1/TAZ activity, on cell migration and invasion in MPMs.

## RESULTS

### RHAMM expression profile varies among MPM cell lines

**Figure 1:**
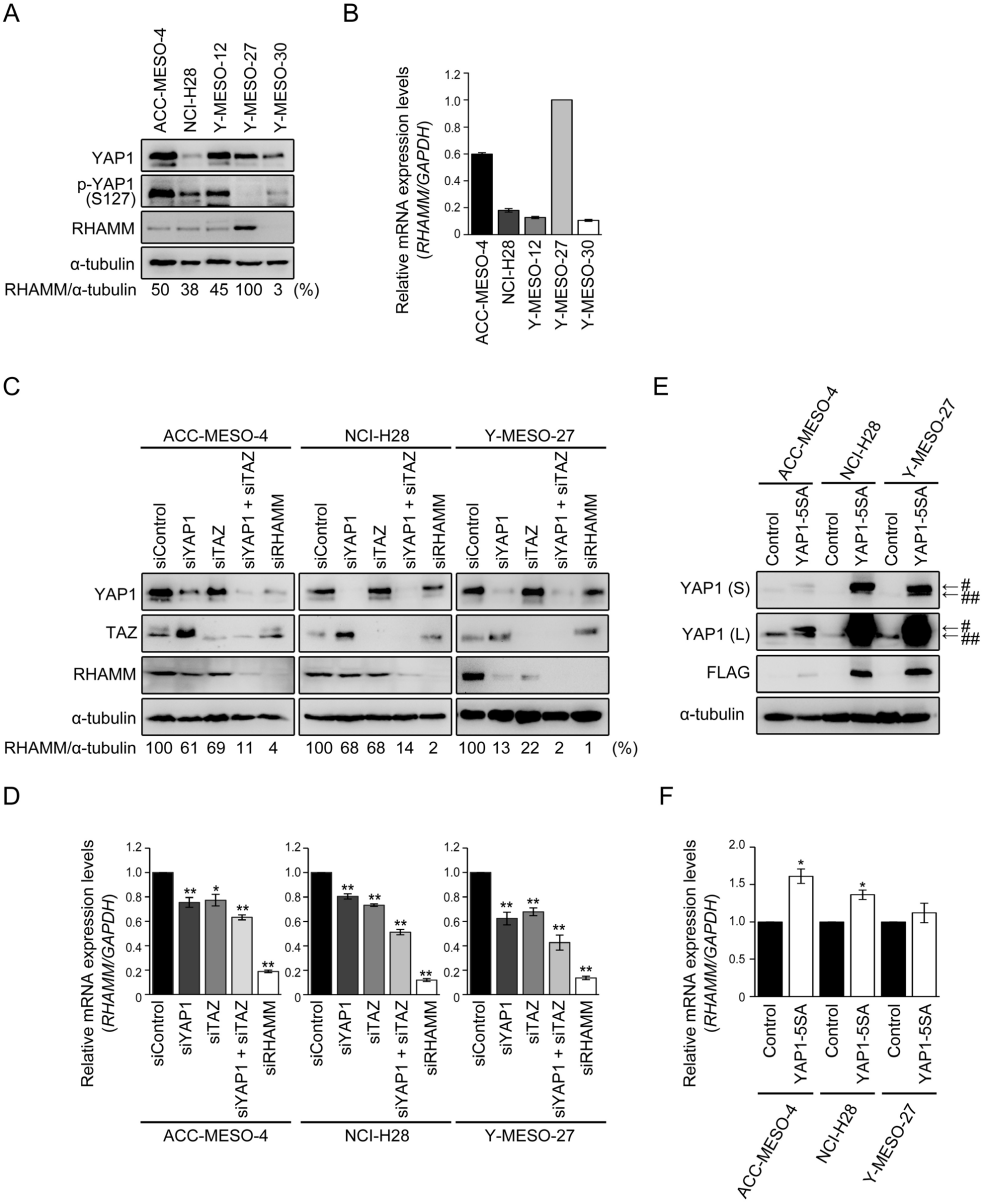
RHAMM expression is regulated by YAP1/TAZ in MPM cells **(A)** Protein and **(B)** mRNA expression profiles of MPM cell lines. Numbers shown in (A) represent values relative to RHAMM/α-tubulin in Y-MESO-27 cells as a reference. **(C)** Protein and **(D)** mRNA expression profiles after single knockdown of YAP1, TAZ, and RHAMM or concomitant knockdown of YAP1 and TAZ in MPM cell lines. Numbers shown in (C) represent values relative to RHAMM/α-tubulin in control siRNA-treated cells as a reference. **(E)** Protein and **(F)** mRNA expression profiles after forced expression of YAP1-5SA in MPM cell lines. “S” and “L” in (E) indicate “short” and “long” exposure, respectively. ^#^ and ^##^ indicate Flag-tagged YAP1 5SA and endogenous YAP1, respectively. Bars in graphs indicate mean ± SEM of three independent experiments. Welch’s t test was used for statistical analysis (^*^P < 0.05, ^**^ *P* < 0.01).

First, we validated the expression levels of YAP1, phosphorylated YAP1 (p-YAP1, S127), and RHAMM in MPM cell lines. The expression profiles of YAP1 and p-YAP1 in all of the cell lines (ACC-MESO-4, NCI-H28, Y-MESO-12, -27, and -30) have been described previously [31]. In this study, we further assessed the relationship with RHAMM expression. Among the cell lines tested, Y-MESO-27 has been shown to harbor a homozygous deletion mutation in *LATS2* gene, a major kinase of Hippo pathway [8]. Because LATS2 phosphorylates serine 127 and causes cytoplasmic sequestration of YAP1, failure of phosphorylation at serine 127 in the YAP1 protein causes constitutive translocation to the nucleus and transcription of its target genes [15, 32]. We confirmed that the phosphorylation level of YAP1 (p-YAP1, S127) in Y-MESO-27 was decreased and that, as expected, RHAMM protein was expressed at high levels (Figure 1A). Although Y-MESO-12 and Y-MESO-30, which harbor a homozygous deletion in *NF2* gene and a partial deletion in *LATS2* gene, respectively [8], were expected to show YAP1 activation, they had lower levels of RHAMM protein (Figure 1A) and mRNA (Figure 1B) than Y-MESO-27. We speculate that an additional mechanism for negative regulation of YAP1, other than the Hippo pathway, may operate in these cell lines. The other cell lines, ACC-MESO-4 and NCI-H28, neither of which harbors mutation in *NF2* and *LATS2* genes, showed relatively low levels of RHAMM protein (Figure 1A) and mRNA (Figure 1B). Based on the genetic background of *Lats2* status described above, we selected three cell lines, ACC-MESO-4 (wild), NCI-H28 (wild), and Y-MESO-27 (*Lats2*-deletion), for further study to investigate the involvement of the YAP1/TAZ-RHAMM axis, as direct regulation of YAP1 by LATS2 might be conserved in these cell lines.

### RHAMM expression is regulated by YAP1/TAZ in MPM cell lines

To address the relationship between YAP1 and RHAMM in MPM cell lines, the small interfering RNA (siRNA) method was employed. TAZ, a paralog of YAP1, has been shown to act redundantly with YAP1 to drive the target gene [33]. Expression of *CTGF* and *ANKRD1*, which are reported to be YAP1/TAZ-regulated genes [32] , was decreased by knockdown of *YAP1* or *TAZ* in the MPM cell lines (Supplementary Figure 1). Concomitant knockdown of *YAP1* and *TAZ* markedly decreased the expression of RHAMM protein in all of the cell lines tested (Figure 1C). As judged by the ratio of RHAMM and α-tubulin, a decrease of over 90% was achieved by concomitant knockdown. Single knockdown of YAP1 or TAZ also modestly decreased RHAMM protein expression in ACC-MESO-4 and NCI-H28 and more significantly in Y-MESO-27 cells (Figure 1C). It is interesting to note that almost complete knockdown of RHAMM (Figure 1C) decreased the cell proliferation of Y-MESO-27 cells only about 50% (Supplementary Figure 3C). These results suggest that the contribution of RHAMM to cell proliferation may be modest. Expression levels of *RHAMM* mRNA were significantly decreased by single or concomitant siRNA treatment (Figure 1D). Consistent results were obtained using other siRNA oligos specifically targeting other sequences in *YAP1* or *TAZ* mRNA (Supplementary Figure 2). Furthermore, forced expression of hyperactive YAP1 (YAP1-5SA, where 5 phosphorylation sites are mutated to alanine, including the major LATS phosphorylation sites) induced *RHAMM* mRNA expression in the MPM cell lines (Figure 1E, 1F). These results indicate that RHAMM expression is regulated by YAP1/TAZ in MPM cell lines.

### Involvement of the YAP1/TAZ-RHAMM axis in cell migration and invasion in MPM cell lines

It has been reported that YAP1 regulates cell proliferation via cell cycle-related gene expression in MPM cell lines [25]. Therefore, we first confirmed the effect of *YAP1/TAZ* knockdown in the MPM cell lines we used. Whereas single knockdown of *YAP1* or *TAZ* did not decrease cell proliferation in our system, concomitant knockdown of *YAP1* and *TAZ* decreased cell proliferation significantly (41%, 43%, and 46% decrease in ACC-MESO-4, NCI-H28, and Y-MESO-27, respectively) (Supplementary Figure 3A).

**Figure 2:**
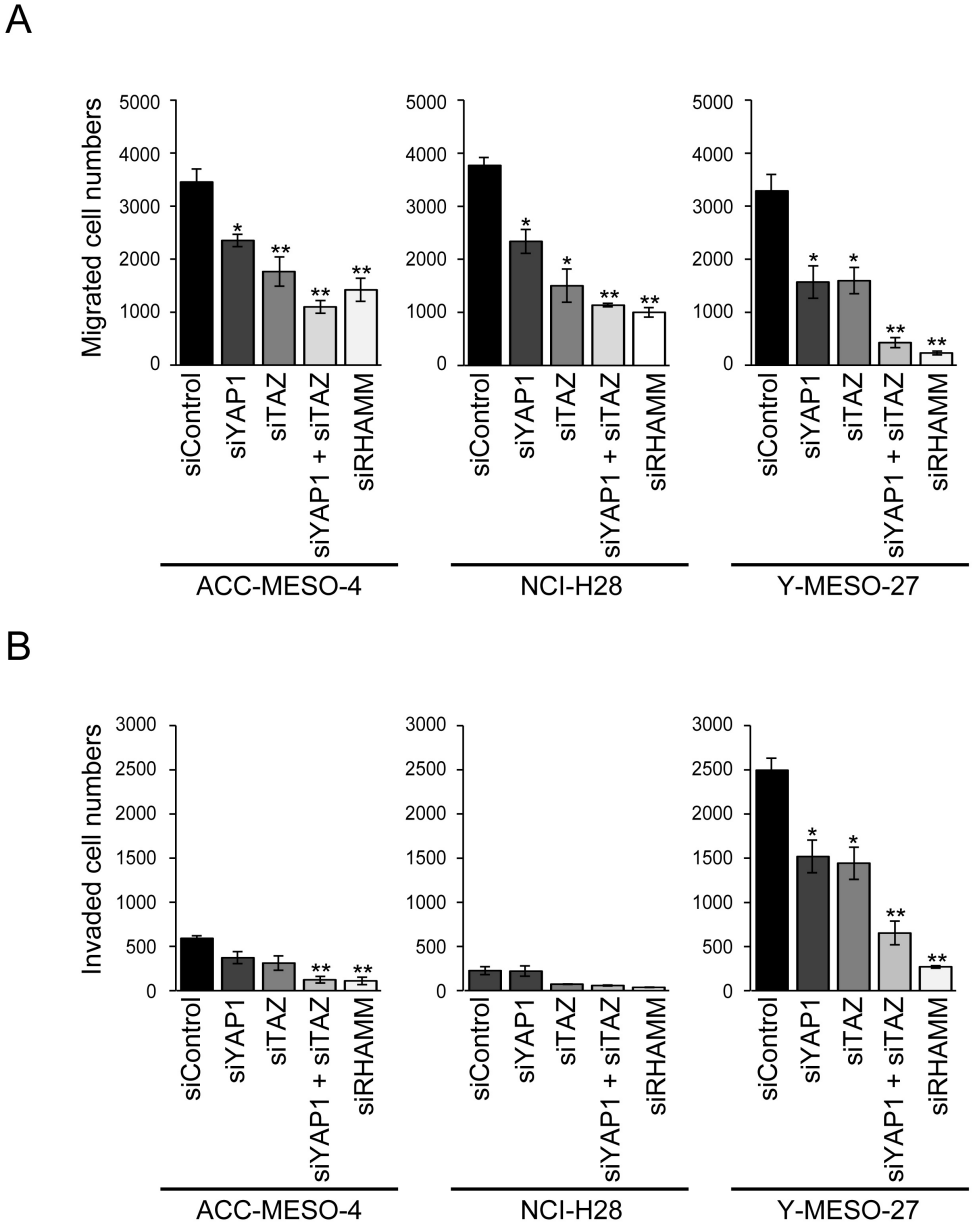
Involvement of the YAP1/TAZ-RHAMM axis in cell migration and invasion in MPM cells **(A)** Migration and **(B)** invasion profiles after single knockdown of YAP1, TAZ, and RHAMM or concomitant knockdown of YAP1 and TAZ in MPM cell lines. Bars in graphs indicate mean ± SEM of three independent experiments. Welch’s t test was used for statistical analysis (^*^P < 0.05, ^**^ *P* < 0.01).

Besides cell proliferation, we hypothesized that YAP1/TAZ-regulated RHAMM might affect cell migration and invasion of the MPM cell lines, since RHAMM has been shown to be involved in cell migration and invasion in various tumors [13, 29, 30]. Therefore, we further studied whether knockdown of *YAP1/TAZ* would affect cell migration and invasion in these cell lines. We found that RHAMM was regulated by YAP1/TAZ (Figure 1) and involved in cell migration (Figure 2A) and invasion (Figure 2B) in the MPM cell lines. Single knockdown of YAP1 or TAZ significantly decreased cell migration in all of the cell lines tested (Figure 2A). Single knockdown of YAP1 or TAZ significantly decreased cell migration, and concomitant knockdown showed a more marked effect on cell migration in all of the cell lines tested (Figure 2A). Similarly, the invasion activities of the cell lines tended to be decreased by YAP1, TAZ, or concomitant knockdown, and RHAMM knockdown also suppressed the invasion activity at or under the same levels with the concomitant knockdown, with Y-MESO-27 (a 74% decrease) showing the most apparent effect (Figure 2B).

These results indicate that the YAP1/TAZ-RHAMM axis significantly contributes the migration and invasion activity of MPM cell lines.

### Stimulation of cell migration and invasion by HA in mesothelioma cell lines is the YAP1/TAZ-RHAMM axis-dependent

RHAMM has been shown to function as a HA receptor and to be involved in cell migration and invasion [13]. Therefore, we assessed whether exogenous HA would stimulate cell migration and invasion in MPM cell lines expressing RHAMM protein (Figure 1).

**Figure 3:**
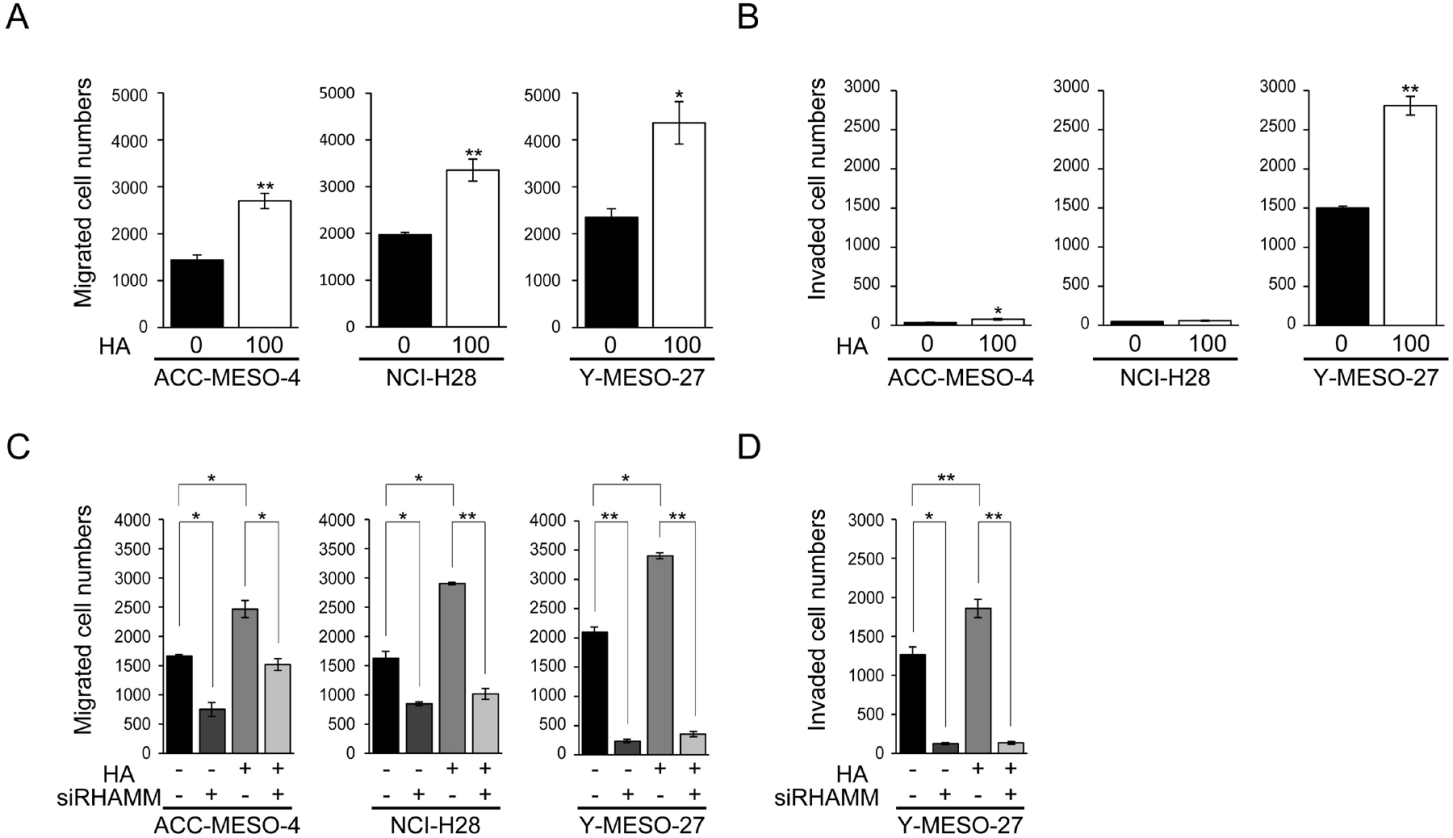
Stimulation of cell migration and invasion by HA in MPM cell lines is the YAP1/TAZ-RHAMM axis-dependent **(A)** Migration and **(B)** invasion profiles after HA treatment. Cells were incubated for 24 h in serum-free medium, and then treated for 24 h with or without 100 μg/ml HA. The cells were then counted, transferred to transwell or matrigel chambers, and cultured for a further 48 h with serum-free medium containing HA. **(C)** Migration and **(D)** invasion profiles after HA treatment in RHAMM-knockdown cells. Cells were transfected with siControl or siRHAMM oligos for 24 h, then counted, transferred to transwell or matrigel chambers, and cultured for a further 48 h with serum-free medium containing HA. Bars in graphs indicate mean ± SEM of three independent experiments. Welch’s t test was used for statistical analysis (^*^P < 0.05, ^**^ *P* < 0.01).

We found that HA significantly stimulated cell migration in all of the cell lines tested (Figure 3A). Cell invasion was stimulated significantly in ACC-MESO-4 and Y-MESO-27 (Figure 3B). To exclude any effect of HA on cell proliferation, we used HA at the concentration (100 μg/ml) that dose not affect cell proliferation significantly (Supplementary Figure 3B)

Next, to further assess whether RHAMM was actually involved in cell migration and invasion by HA, RHAMM knockdown experiments were performed. RHAMM knockdown without HA treatment decreased cell migration significantly (Figure 2A, 3C). As expected, the increase of cell migration induced by HA was significantly decreased by RHAMM knockdown in all of the cell lines tested (Figure 3C). The same trend was observed for cell invasion in Y-MESO-27 (Figure 3D). These results indicated that exogenously applied HA stimulated cell migration and invasion through RHAMM in these MPM cell lines.

### Statin treatment decreases RHAMM-dependent cell migration and invasion via YAP1/TAZ regulation in MPM cell lines

**Figure 4:**
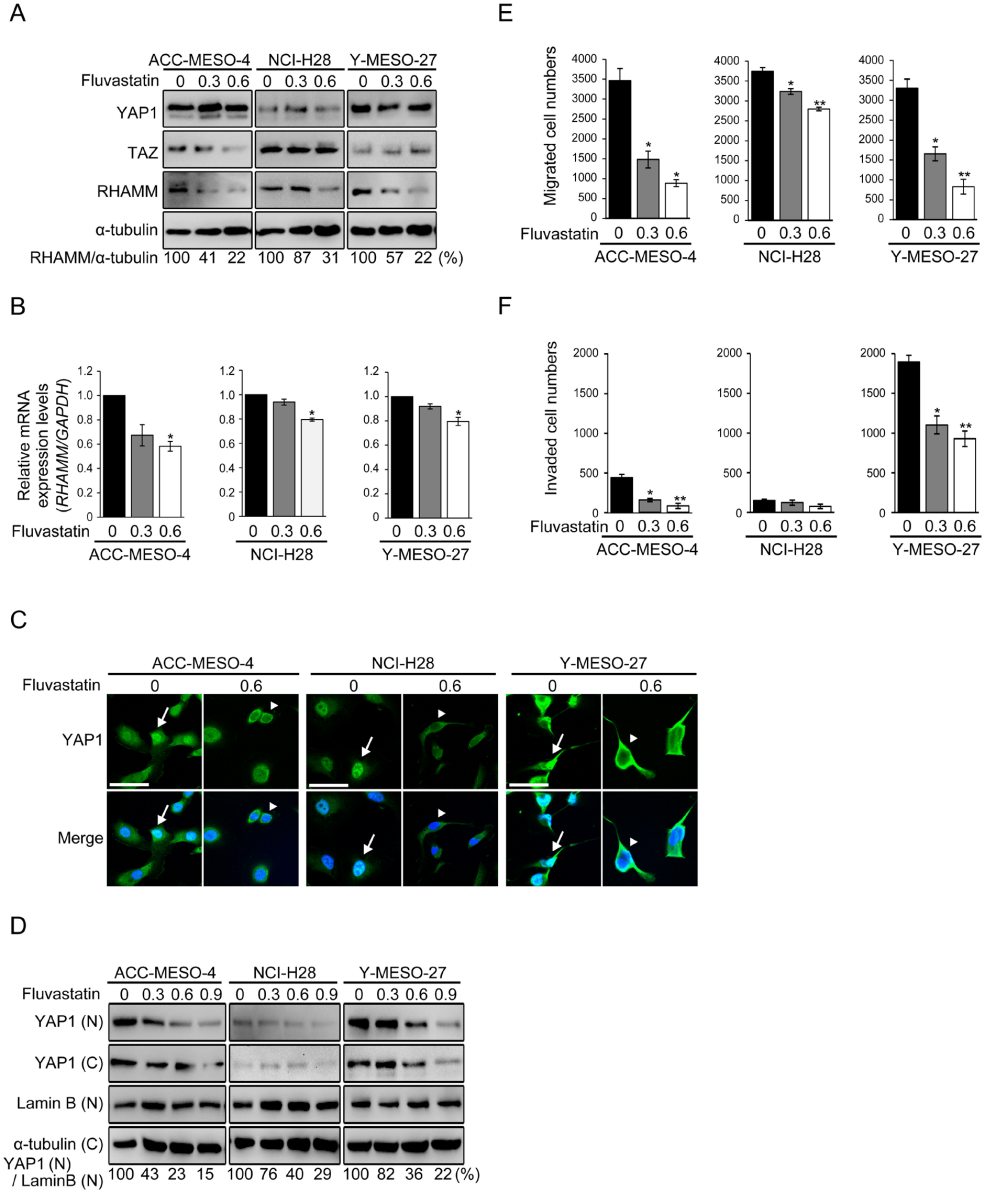
Statin treatment decreases RHAMM-dependent cell migration and invasion via YAP1/TAZ regulation in MPM cell lines **(A)** Protein and **(B)** mRNA expression profiles after treatment with fluvastatin. Cells were incubated for 48 h with 0, 0.3 or 0.6 μM fluvastatin. **(C)** Cellular localization of YAP1 in fluvastatin-treated MPM cell lines. Cells were treated with or without 0.6 μM fluvastatin for 48 h. Arrows indicate nuclear translocation and arrowheads indicate cytoplasmic sequestration of YAP1. Scale bar represents 50 μm. **(D)** Western blot analysis of the nuclear and cytoplasmic fractions. Cells were treated with fluvastatin at 0, 0.3, 0.6 or 0.9 μM for 48 h. “N” and “C” indicate the nuclear and cytoplasmic fractions, respectively. Numbers below represent values relative to YAP1(N)/Lamin B(N) in treated with 0 μM fluvastatin cells as a reference. **(E)** Migration and **(F)** invasion profiles after fluvastatin treatment. Cells were incubated with 0, 0.3 or 0.6 μM fluvastatin for 24 h in medium containing 10% FBS, and then incubated in transwell or matrigel chambers for 48 h. Bars in graphs indicate mean ± SEM of three independent experiments. Welch’s t test was used for statistical analysis (^*^P < 0.05, ^**^ *P* < 0.01).

Statins, which are 3-hydroxy-methylglutaryl CoA reductase inhibitors generally used as cholesterol-lowering drugs, are also reported to inhibit the activity of YAP1 in breast cancer [13]. Here, we investigated whether fluvastatin, a member of the statin drug class, was able to decrease cell migration and invasion by inhibiting the YAP1/TAZ-RHAMM axis. First, we assessed the expression profile of RHAMM protein after fluvastatin treatment in the MPM cell lines. The concentration of fluvastatin used in this experiment (maximum 0.6 μM) did not significantly affect cell proliferation (Supplementary Figure 3C). A dose-dependent decrease of RHAMM expression by fluvastatin was observed in all of the cell lines (Figure 4A). Similar trends were observed for mRNA (Figure 4B). Other YAP1-regulated genes such as *CTGF* and *ANKRD1* were also significantly inhibited by fluvastatin treatment (Supplementary Figure 4). Next, we assessed the cellular localization of YAP1 protein using immunofluorescence, as fluvastatin has been shown to suppress YAP1 activity by inhibiting the nuclear localization of YAP1 protein [34]. As expected, fluvastatin treatment caused cytoplasmic sequestration of YAP1 in all of the MPM cell lines (Figure 4C). For confirmation, we also performed a Western blotting analysis using the nuclear/cytoplasmic fraction (Figure 4D) and observed a dose-dependent inhibitory effect of fluvastatin on YAP1 translocation. It is worth noting that fluvastatin at 0.6 μM, which was the maximum concentration we used for the proliferation, migration and invasion assay, inhibited the nuclear translocation of YAP1 by only 55 77 %, 39 60 % and 22 64 % in ACC-MESO-4, NCI-H28 and Y-MESO-27 cells, respectively (Figure 4D). We speculate that this partial incomplete inhibition might have accounted for the less inhibitory effect of fluvastatin on cell proliferation (Supplementary Figure 3C).However, a significant decrease of cell migration was observed in all of the cell lines (Figure 4E), and cell invasion was also reduced, except in NCI-H28 (Figure 4F), at this concentration.

**Figure 5:**
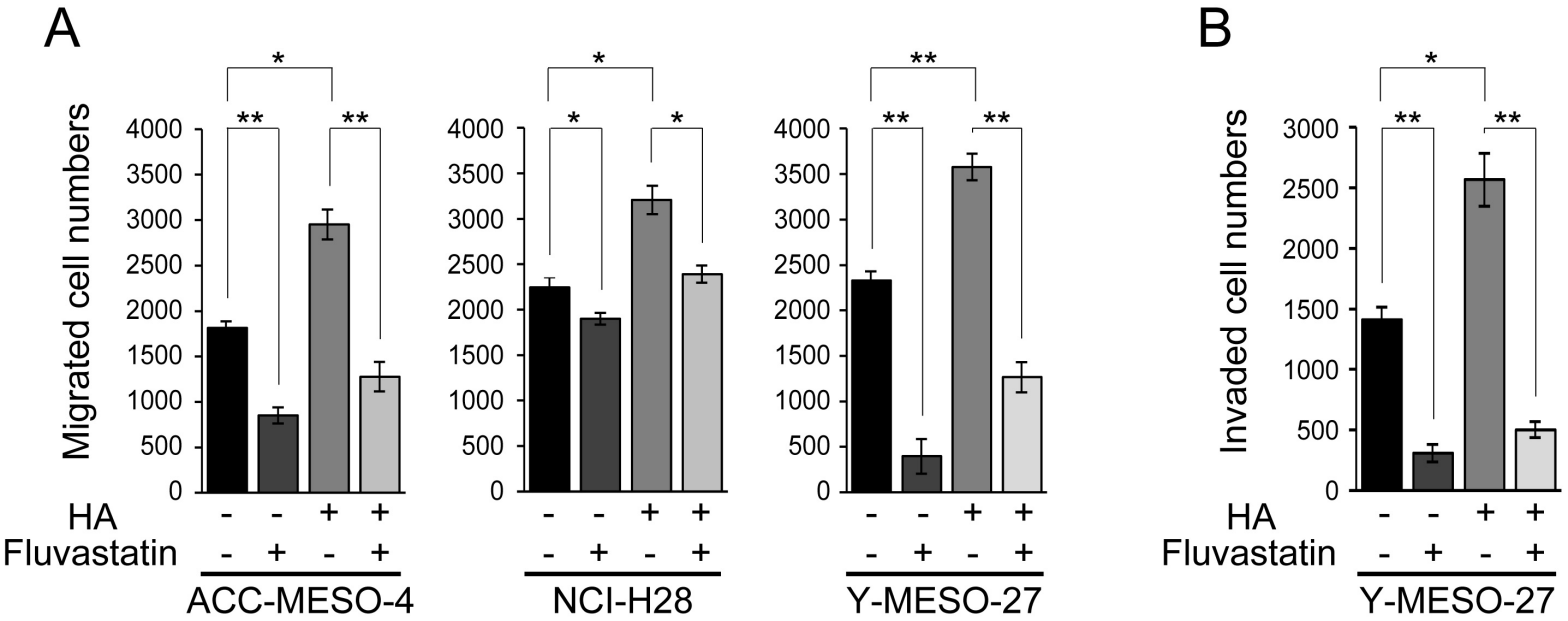
Fluvastatin inhibits HA-augmented cell migration and invasion in MPM cell lines **(A)** Migration and **(B)** invasion profiles after concomitant treatment with HA and fluvastatin. Cells were incubated for 24 h in serum-free medium, treated for 24 h with 0.6 μM fluvastatin with or without 100 μg/ml HA, and then cultured further for 48 h. Bars in graphs indicate mean ± SEM of three independent experiments. Welch’s t test was used for statistical analysis (^*^P < 0.05, ^**^ *P* < 0.01).

Finally, we applied fluvastatin to HA-treated MPM cell lines and assessed its inhibitory effect on cell migration and invasion. Fluvastatin significantly decreased cell migration in all of the MPM cell lines (Figure 5A) and also decreased cell invasion in Y-MESO-27 (Figure 5B). These results indicate that fluvastatin treatment inhibits YAP1/TAZ activity and decreases RHAMM-dependent cell migration and invasion in MPM cell lines.

## DISCUSSION

YAP1/TAZ have various roles in the regulation of homeostasis, and their dysregulation is associated with tumorigenesis and cancer progression [33]. In the present study, we found that the YAP1/TAZ-RHAMM axis is involved in the regulation of migration and invasion in MPM cell lines, and that HA, which is massively present in pleural effusion in MPM patients, may contribute to the progression of mesothelioma. Moreover, fluvastatin, which has been generally used to treat hypercholesterolemia and recently has also been shown to module YAP1/TAZ activity, effectively decreased cell migration and invasion in these MPM cell lines. Although recent studies have revealed the contributions of YAP1/TAZ to cell proliferation in MPMs [31] , our study has additionally revealed their role in cell migration and invasion.

LATS1/2 is the major kinase that directly phosphorylates YAP1 at five serine residues, and TAZ has four of these sites [15]. It has been reported that the frequency of *LATS2* alterations in MPMs is around 20% [8]. YAP1/TAZ, which are downstream effectors of LATS1/2, act as transcriptional co-activators and regulate various target genes [35]. Among them, we focused on the expression of RHAMM, a HA receptor known to be involved in cell migration and invasion [36]. RHAMM is expressed at low levels in most normal tissues and up-regulated during wound repair in response to hypoxia and growth factors, hyper-expression of RHAMM being associated with tumor development, progression, and metastasis [37]. Bifunctionally, RHAMM has also been shown to regulate mitotic-spindle integrity and to be involved in cell proliferation [38]. Our results indicated that although knockdown of RHAMM decreased cell proliferation significantly (Supplementary Figure 3A), the resulting effects on cell migration (Figure 2A) and invasion (Figure 2B) were more potent in the MPM cell lines we tested. Notably, MPM patients with higher expression of RHAMM had a significantly worse prognosis compared with low-expression (Supplementary Figure 5). A recent study has revealed that one of the HA receptors, CD44, is regulated by YAP1/TAZ in MPMs [20]. Interestingly, extracellular HA binding to CD44-RHAMM complexes confers malignant potential in breast cancer [39]. As a downstream effect, RHAMM has been shown to activate the ERK signaling pathway [40] and recently ERK activation by RHAMM was also shown to be involved in cell migration and invasion [13]. Taken together, our results suggested that RHAMM itself, or the mechanism responsible for regulating its expression, might be an attractive target for MPM therapy.

We observed evident differences in invasion activity between the MPM cell lines (Figure 2B). A recent study has indicated that LATS2 negatively regulates the expression of matrix metalloproteinase-2 and -9 in non-small cell lung cancer (NSCLC) [41]. Metalloproteinases are required for cell invasion because they degrade various cell adhesions and matrices [42]. The present results suggest that MPMs harboring *LATS2* mutation may show relatively higher frequencies of metastasis to intra-thoracic lymph nodes.

Our study revealed that concomitant knockdown of both YAP1 and TAZ had a greater effect on cell migration and invasion than single knockdown of either (Figure 2). This may be because YAP1 and TAZ may act redundantly to regulate target genes such as *CTGF*, *ANKRD1*, and *RHAMM* in MPM cell lines (Figure 1C, 1D). Interestingly, the expression level of TAZ protein was increased to a greater degree by single knockdown of YAP1 in ACC-MESO-4 and NCI-H28 cells, which do not harbor *LATS2* mutation (Figure 1C). These results are consistent with a previously reported study indicating that cells have an intrinsic mechanism for maintaining total output/activity of Hippo pathway [43].

Finally, a high concentration of HA in pleural effusion is one of the most notable diagnostic features of MPM patients [26]. It has been reported that hyaluronan fragments induce cytokines and metalloproteinase partly through Toll-like receptor 4 signaling [44] , suggesting that HA promotes inflammation. Furthermore, HA promotes the progression of various digestive tract cancers [45] and pancreatic cancer [46]. Here we observed that treatment of MPM cell lines with HA enhanced cell migration and invasion (Figure 3A), suggesting HA in the pleural effusion of MPM patients may promote malignancy via RHAMM. Therefore, RHAMM or its regulatory factors such as YAP1/TAZ may represent attractive therapeutic targets for MPM.

Because MPM is one of the most aggressive forms of tumor, showing resistance to various forms of chemotherapy, the development of novel therapeutic strategies has been anticipated. In conclusion, our present results and the available data fully support the possibility that decreased expression or inhibition of YAP1/TAZ or RHAMM may provide a novel avenue for treatment for patients with MPM in order to inhibit progression of the disease.

## MATERIALS AND METHODS

### Cell culture and transfection

Three human MPM cell lines (Y-MESO-12, Y-MESO-27, and Y-MESO-30) were established in Aichi Cancer Center Research Institute (Nagoya, Aichi, Japan) [31, 47]. One human MPM cell line (ACC-MESO-4) was also obtained from the Riken Cell Bank (Tsukuba, Ibaraki, Japan) and another (NCI-H28) from the American Type Culture Collection (Manassas, VA, USA). All of these MPM cell lines were cultured at 37°C under 5% CO_2_ in RPMI 1640 medium (Thermo Fisher Scientific, Waltham, MA, USA) supplemented with 10% fetal bovine serum (FBS) and antibiotics. The pCMV-flag YAP 5SA plasmid (Deposited by Dr. Guan K. Lab, #27371) was obtained from Addgene (Cambridge, MA, USA). Cells were transiently transfected using Lipofectamine 3000 Reagent (Thermo Fisher Scientific) in Opti-MEM I Reduced Serum Medium (Thermo Fisher Scientific) for 48 h and assayed.

### Reagents and antibodies

Fluvastatin sodium (PHR1620) was obtained from Sigma Aldrich (St Louis, MO, USA). Hyaluronic acid (Molecular Weight: 15-40 kDa) (GLR001) was obtained from R&D Systems (Minneapolis, MN, USA). Primary antibodies against YAP (#14074, 1:100 for immunofluorescence or 1:1000 for Western blotting; Cell Signaling Technology, Danvers, MA, USA), p-YAP (#13008, 1:1000 for Western blotting; Cell Signaling Technology), TAZ (560235, 1:500 for Western blotting; BD Pharmingen, Franklin Lakes, NJ, USA), RHAMM (CD168) (ab170527, 1:250 for Western blotting; Abcam, Cambridge,UK), α-tubulin (T5168, 1:4000 for Western blotting; Sigma Aldrich), and FLAG M2 (F3165, 1:1000 for Western blotting; Sigma Aldrich), and DAPI solution (D523, 1:1000 for nuclear staining, Dojindo Laboratories, Kumamoto, Japan) were used in accordance with the manufacturer’s instructions. Anti-Rabbit IgG, HRP-Linked Whole Ab Donkey (NA934), and Anti-Mouse IgG, HRP-Linked Whole Ab Sheep (NA931) were obtained from GE Healthcare (Little Chalfont, Buckinghamshire, UK).

### Western blotting

Cells were washed twice with cold 0.15 M NaCl, fixed in 0.15 M NaCl with 10% trichloroacetic acid, and incubated overnight at 4°C. Total protein was extracted in 9M urea with 2% Triton X-100 (Thermo Fisher Scientific) and quantified using a BCA Protein Assay Kit (Thermo Fisher Scientific). Proteins were separated on 10% SDS-PAGE gels and transferred to FluoroTrans W Membranes (Pall Corporation, Port Washington, NY, USA). The membranes were then blocked with 5% dry milk and incubated overnight at 4°C with each of the primary antibodies. After washing, the membranes were incubated for 2 h at room temperature in 3% bovine serum albumin (BSA) (Sigma Aldrich) with secondary antibodies. Signals were detected with ECL Prime Western Blotting Detection Reagent (GE Healthcare) and ChemiDoc XRS (Bio-Rad Laboratories, Hercules, CA, USA).

### Quantitative real-time PCR

Total RNA was extracted using TRIzol Reagent (Thermo Fisher Scientific) in accordance with the manufacturer’s instructions. RNA was reverse-transcribed using a SuperScript III First-Strand Synthesis System (Thermo Fisher Scientific) in accordance with the manufacturer’s instructions. Real-time PCR was performed using an Applied Biosystems 7500 Real-Time PCR System (Thermo Fisher Scientific) and TaqMan Gene Expression MasterMix and TaqMan Gene Expression Assays (Thermo Fisher Scientific).

### Small-interfering RNA transfection

Silencer Select siRNAs against YAP1 (Cat.; # 4392420, ID; s20367), TAZ (WWTR1) (Cat.; # 4392420, ID; s24787), RHAMM (HMMR) (Cat.; # 4390824, ID; s6671), and negative control siRNA (Cat.; # 4390844) were obtained from Thermo Fisher Scientific. Cells were treated with 10 nM siRNA in the presence of Lipofectamine RNAiMAX Transfection Reagent (Thermo Fisher Scientific) for 48 h.

### Cell migration and invasion assays

For transwell migration assays, 2×10^4^ cells were placed in the top chamber on a non-coated membrane (24-well insert; pore size, 8 μm; BD Bioscience, San Jose, CA, USA). For invasion assays, 2×10^4^ cells were placed in the top chamber on a Matrigel-coated membrane (24-well insert; pore size, 8 μm; BD Bioscience). In both assays, the cells were placed in serum free medium, and medium supplemented with 20% FBS was used in the lower chamber. The cells were incubated for 48 h, and those that did not migrate or invade through the pores were removed with a cotton swab. The cells were fixed with methanol, stained with DAPI (Dojindo Laboratories, Kumamoto, Japan), and then counted using a BZ-9000 BioRevo (KEYENCE, Elmwood Park, NJ, USA) and ImageJ software (open resource software obtained from https://imagej.nih.gov/ij/).

### Immunofluorescence staining

Two thousand cells were cultured for 48 h on poly-L-lysine-coated cover slips in 6-well plates. Cells were fixed with 4% paraformaldehyde for 30 min at room temperature and blocked for 2 h in blocking buffer (1 × PBS with 0.3% Triton X-100 and 5% Goat serum (Sigma Aldrich)). They were then incubated overnight at 4°C with 1 × PBS and 0.3% Triton X-100, 1% BSA and anti-YAP antibody (1:100). Goat anti-Rabbit IgG cross-adsorbed secondary antibody, Alexa Fluor 488 (Thermo Fisher Scientific), was applied for 2 h at room temperature in 1 × PBS with 0.3% Triton X-100 and 1% BSA. Nuclei were stained with DAPI (1:1000) for 5 min on ice. Cells were mounted with Prolong Gold antifade reagent and observed using a confocal microscope (C1 and EZ-C1, Nikon, Tokyo, Japan).

### Subcellular fractionations

An NE-PER Nuclear and Cytoplasmic Extraction Reagents Kit (Thermo Fisher Scientific) was used for nuclear and cytoplasmic isolation. One million cells were used for subcellular fractionation in accordance with the manufacturer’s instructions.

### Statistical analysis

Data are presented as mean ± SEM. Significance of differences was analyzed using Welch’s t test. *P* < 0.05 was considered to indicate statistical significance.

## SUPPLEMENTARY MATERIALS FIGURES



## Abbreviations

ANKRD1: ankyrin-repeat domain containing 1
CTGF: connective tissue growth factor
HA: hyaluronic acid
MPM: malignant pleural mesothelioma
RHAMM: receptor for hyaluronic acid-mediated motility
TAZ: transcriptional co-activator with PDZ-binding motif
YAP1: Yes-associated protein 1
