# Supplementary material for: Hyaluronic acid enhances cell migration and invasion via the YAP1/TAZ-RHAMM axis in malignant pleural mesothelioma
Source: Oncotarget. 2017 Sep 8;8(55):93729–40. doi: 10.18632/oncotarget.20750 (PMC5706831; doi:10.18632/oncotarget.20750)
Supplement: Supplementary file 1 [file oncotarget-08-93729-s001.pdf]

Hyaluronic acid enhances cell migration and invasion via the YAP1/TAZ-RHAMM axis in malignant pleural mesothelioma

SUPPLEMENTARY MATERIALS

Small-interfering RNA oligos

Silencer Select siRNAs against YAP1 #2 and #3 (Cat.; # 4392420, ID; s20366 and s20368) and TAZ (WWTR1) #2 and #3 (Cat.; # 4392420, ID; s24788 and s24789) were obtained from Thermo Fisher Scientific.

Cell proliferation assay

A Cell Counting Kit-8 (Dojindo Laboratories), which utilizes water-soluble tetrazolium salts, was used in accordance with the manufacturer's protocol to evaluate the proliferation of MPM cell lines after treatment with siRNA, HA or fluvastatin. For HA or fluvastatin treatment, cells were seeded into 96-well plates (5×10^4 cells/well) and cultured for 24 h prior to treatment. After treatment with HA or fluvastatin for 48 h, the medium in each well was replaced with 100 µl of drug-free fresh medium and 10 µl of Cell Counting Kit-8 solution, incubation was performed for an additional 1–2 h, and the absorbance of each well at 450 nm was then measured using a Multiskan Spectrum spectrophotometer (Thermo Fisher Scientific).

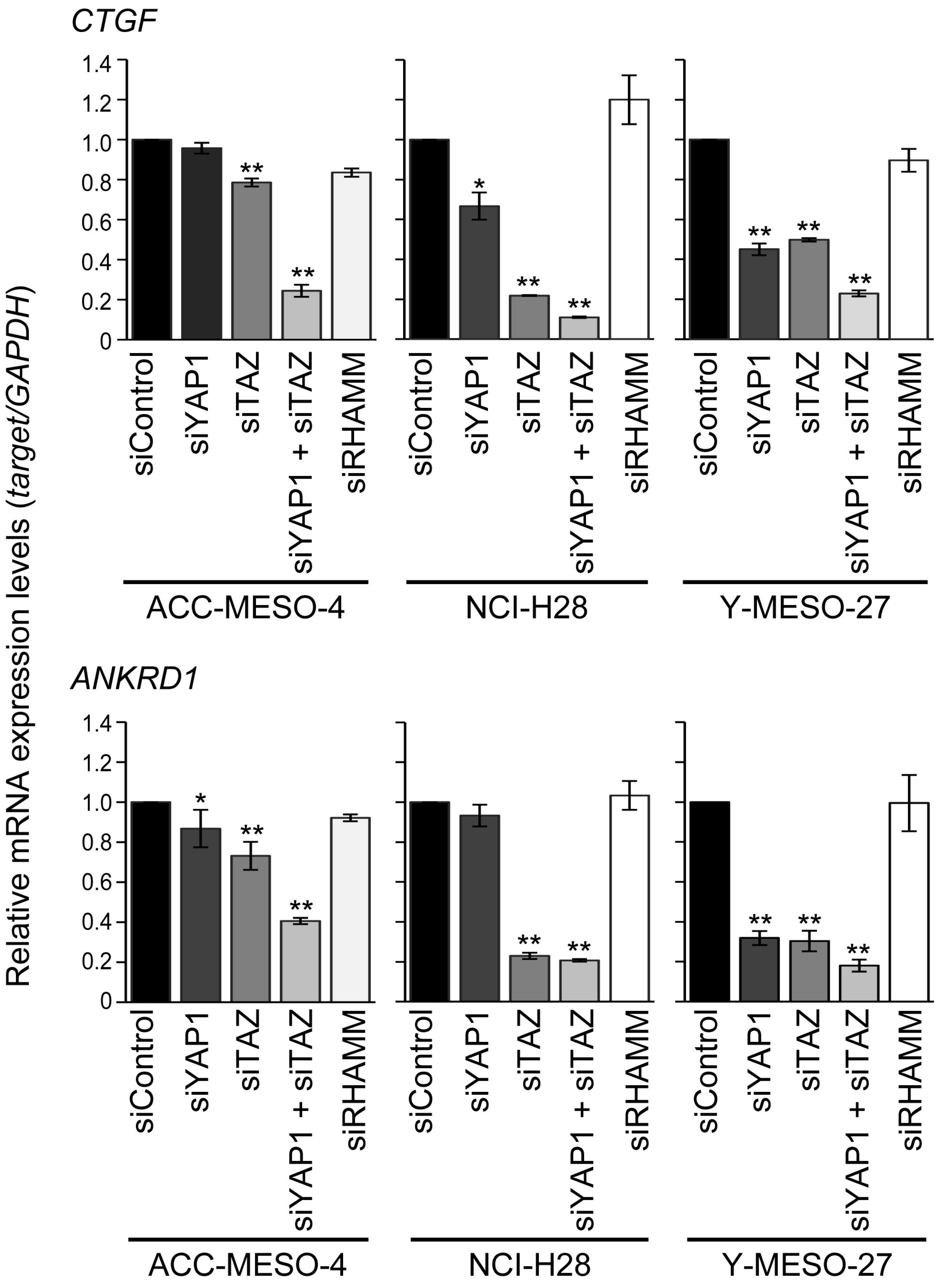

Supplementary Figure 1: Effect of YAP1/TAZ knockdown on other target genes in MPM cells. The CTGF (upper panels) and ANKRD1 (lower panels) mRNA expression profiles after single knockdown of YAP1, TAZ, and RHAMM or concomitant knockdown of YAP1 and TAZ in MPM cell lines were shown. Bars in graphs indicate mean ± SEM of three independent experiments. Welch's t test was used for statistical analysis ($^{*}p<0.05$, $^{**}p<0.01$).

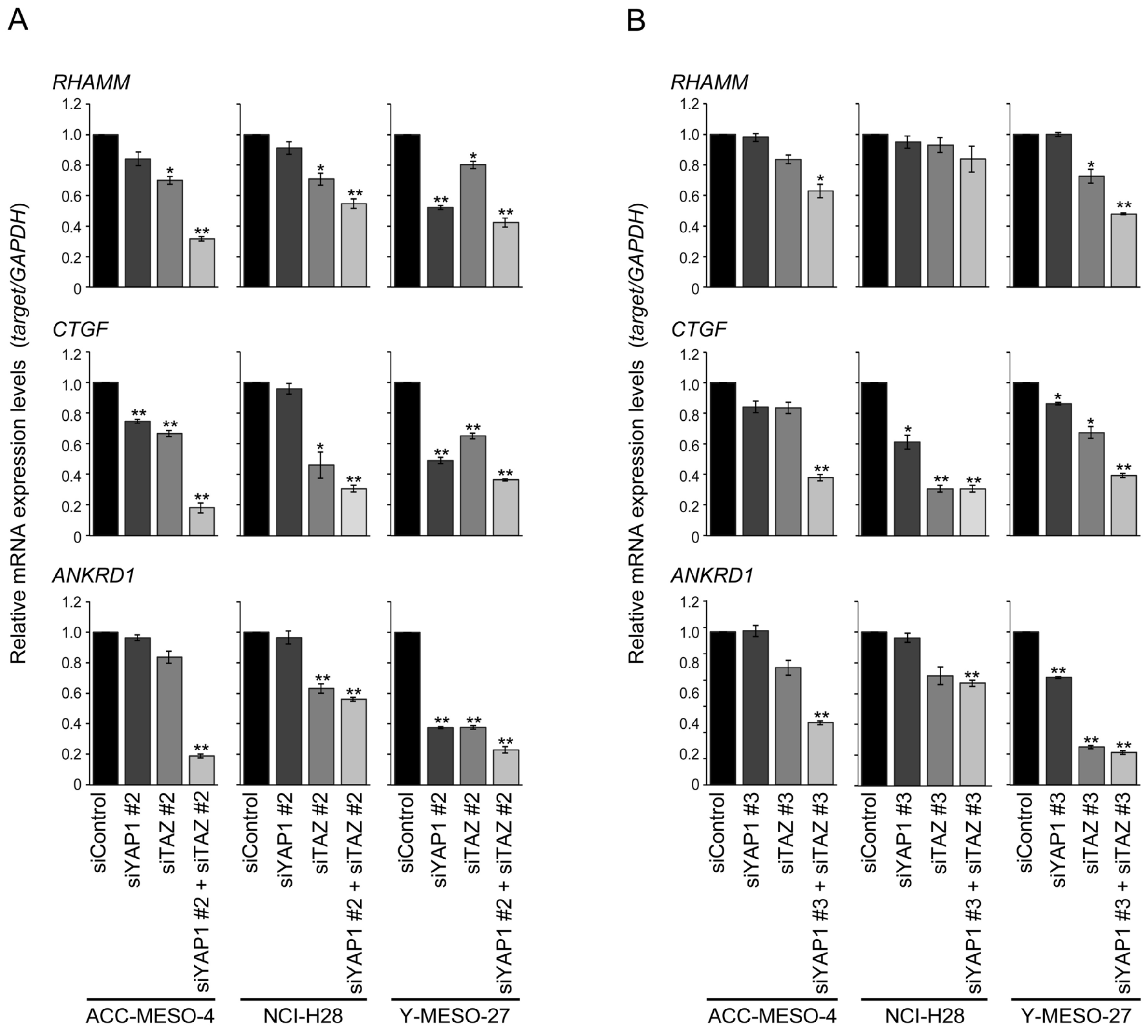

Supplementary Figure 2: Effect of other siRNA oligos specifically targeting other sequences in YAP1 or TAZ mRNA in MPM cells. The RHAMM (upper panels), CTGF (middle panels) and ANKRD1 (Iower panels) mRNA expression profiles after single knockdown of YAP1, TAZ or concomitant knockdown of YAP1 and TAZ in MPM cell lines were shown. **(A)** #2 siRNA and **(B)** #3 siRNA, respectively. Bars in graphs indicate mean ± SEM of three independent experiments. Welch's t test was used for statistical analysis ($^{*}p<0.05$, $^{**}p<0.01$).

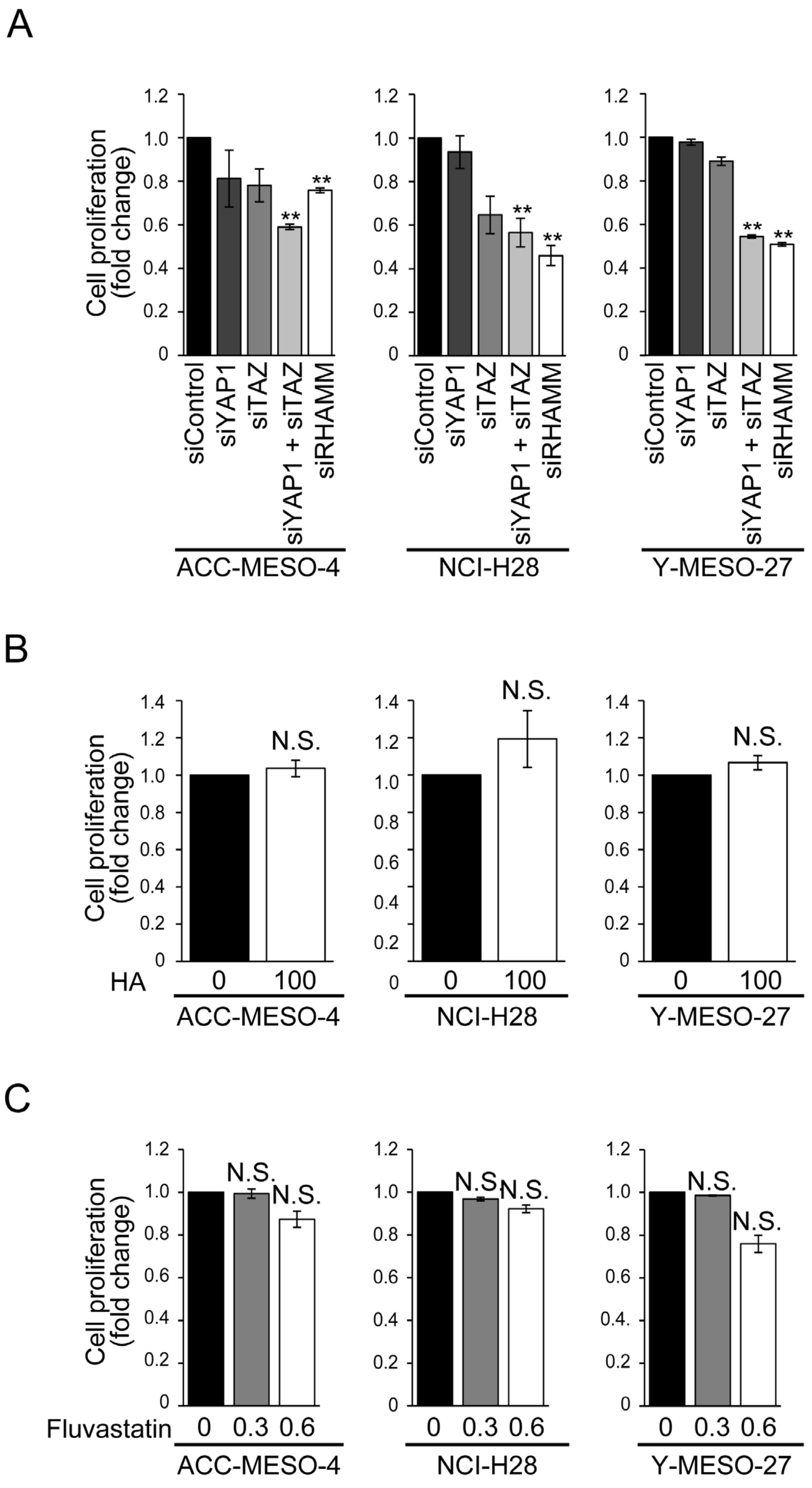

Supplementary Figure 3: Effect of siRNA, HA and fluvastatin treatment on cell proliferation in MPM cells. The effect of **(A)** siRNA **(B)** HA treatment and **(C)** fluvastatin treatment on cell proliferation in MPM cell lines were shown. Cells were incubated for 24 h in serum-free medium, treated for 24 h with 0.6 µM fluvastatin with or without 100 µg/ml HA, and then cultured further for 48 h. Bars in graphs indicate mean ± SEM of three independent experiments. Welch's t test was used for statistical analysis ($^{**}p<0.01$). N. S. not significant.

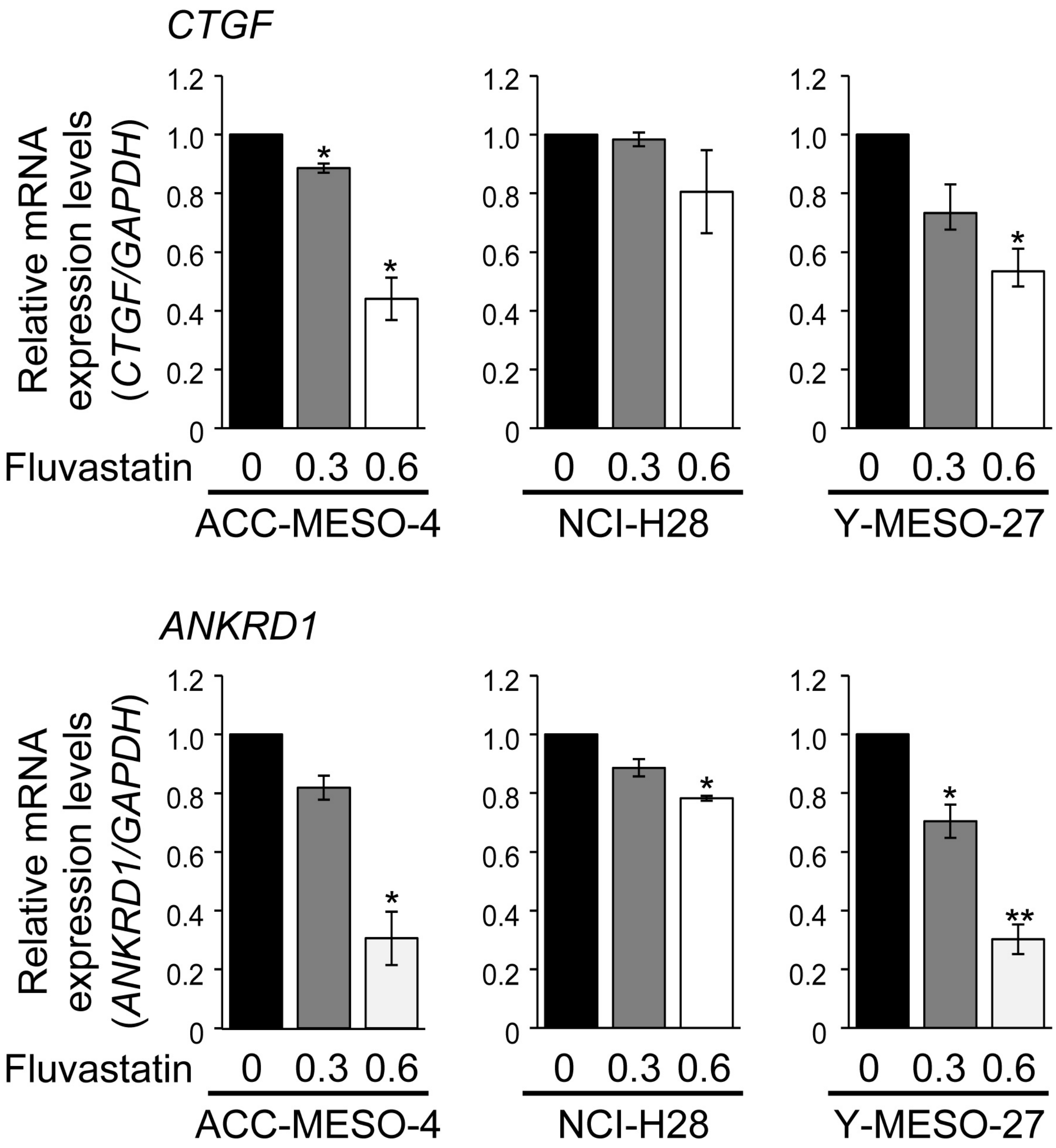

Supplementary Figure 4: Effect of fluvastatin on other YAP1/TAZ target genes in MPM cells. The CTGF (upper panels) and ANKRD1 (lower panels) mRNA expression profiles after treatment with fluvastatin in MPM cell lines were shown. Cells were incubated for 48 h with 0, 0.3 or 0.6 μM fluvastatin. Bars in graphs indicate mean ± SEM of three independent experiments. Welch's t test was used for statistical analysis ($^{*}p<0.05$, $^{**}p<0.01$).

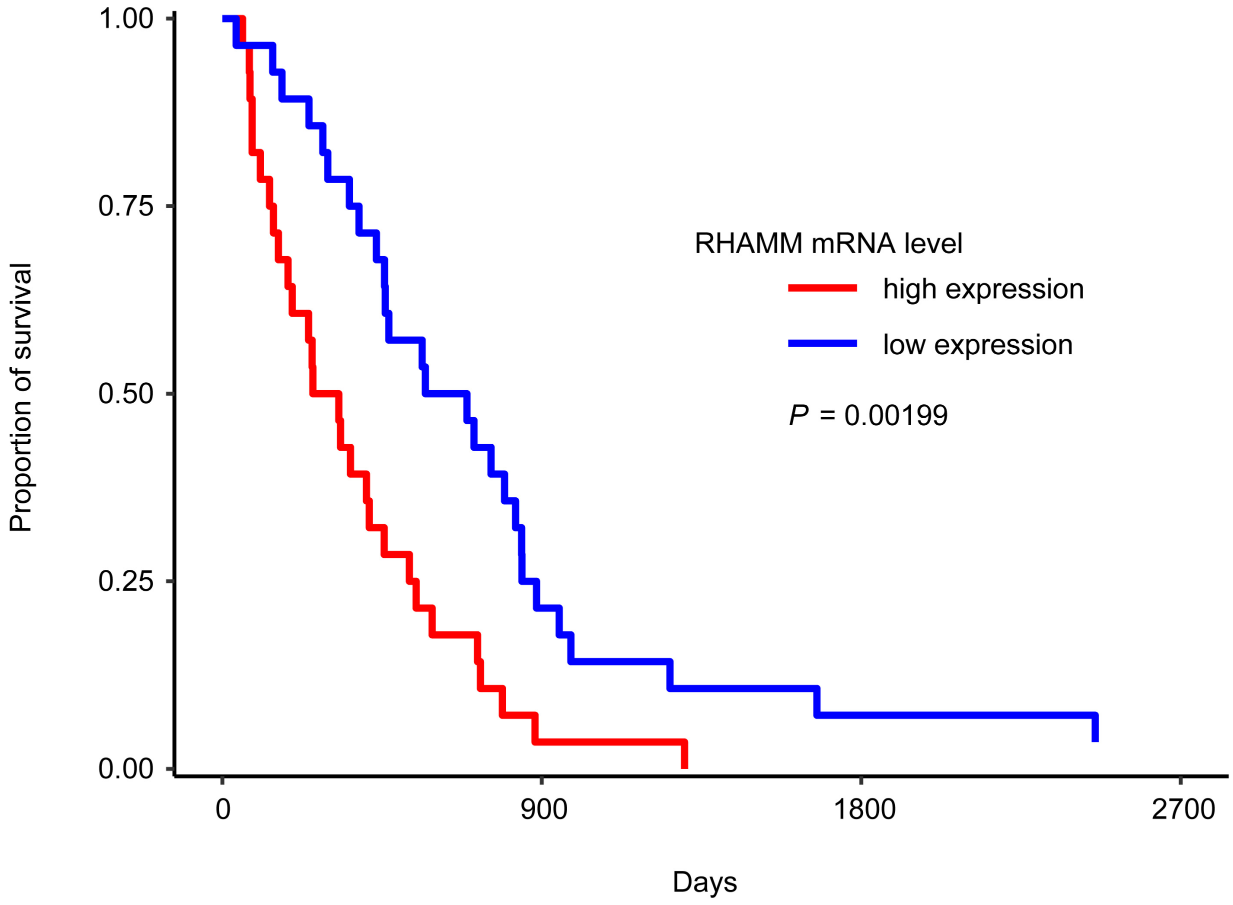

Supplementary Figure 5: Kaplan-Meier survival analysis in MPM patients. Patient information of malignant mesothelioma used in this study was collected from the TCGA website (https://cancergenome.nih.gov/). Kaplan-Meier survival curves were generated for 56 patients whose survival date from their initial pathologic diagnosis date was registered. These patients were divided into two groups based on the median value of their RHAMM mRNA expression level.
